# Facilitating Uptake of Post-abortion Contraception for Young People in Kenya

**DOI:** 10.3389/fgwh.2021.733957

**Published:** 2022-01-20

**Authors:** Faith Mbehero, Ruth Momanyi, Kate Hesel

**Affiliations:** ^1^Clinical Quality of Care Consultant: Planned Parenthood Global, Nairobi, Kenya; ^2^Program Learning Officer, Planned Parenthood Global, Africa Regional Office, Nairobi, Kenya; ^3^Director of Program Learning and Impact, Planned Parenthood Global, New York, NY, United States

**Keywords:** post abortion care, contraception, abortion, young people, long acting reversible contraception (LARC), community engagement, post-abortion contraception and family planning

## Abstract

Globally, maternal mortality is unacceptably high, and unsafe abortion is the most easily preventable cause of maternal death. Post-abortion contraception, recognized as a High Impact Practice in Family Planning, can reduce rates of unintended pregnancies and unsafe abortion and ultimately save lives. Implementation of this, however, is limited, especially for young people. This case study documents strategies, results, and lessons learned from Planned Parenthood Global's project in South West Kenya, which improved access to and provision of comprehensive abortion care, including safe abortion, post-abortion care and post-abortion contraception, at 80 public and private health facilities. By prioritizing training and mentorship of mid-level providers on both medical and surgical abortion care, post-abortion contraception and youth friendly services, in addition to community engagement and referrals, this intervention removes common barriers to care for women and young people. Eighty-five percent of abortion care clients served by the project accepted same-day contraception, with the vast majority—including 90% of clients aged 24 and under—choosing long-acting reversible contraception. The Closing the Gap project was funded by an anonymous donor.

## Introduction

Globally, maternal mortality is unacceptably high. In 2017, an estimated 295,000 women died during and following pregnancy and childbirth (1). The vast majority of these deaths (94%) occurred in low-resource settings, with Sub-Saharan Africa accounting for roughly two-thirds (196,000) of these deaths. Complications of abortion are the fourth leading direct cause of maternal mortality (7.9%), likely an underestimation because of underreporting and misclassification of abortion-related deaths (2). Globally, over 55 million abortions occur annually; 76% of those in sub-Saharan Africa are unsafe (3). An abortion is classified as safe if it takes place using a safe method and is done by an appropriately trained provider (i.e., per WHO health worker guidelines) (4). Less-safe abortions are those that meet only one of the two criteria, and least-safe abortions are those that meet neither. An estimated 50% of unsafe abortions in Africa occur among young women aged 15–24 years (5).

Unsafe abortion is the most easily preventable cause of maternal death. Access to comprehensive abortion care is critical; first trimester abortion, when most abortions are performed, can be safely performed by trained mid-level providers in primary care health facilities (6). Post-abortion care (PAC) is a package of interventions, including the treatment of complications of miscarriage and induced abortion, that reduces abortion-related deaths and disability (7). A critical component of abortion care is the provision of voluntary contraceptive counseling and services to reduce unintended pregnancies and repeat abortions (2). Contraceptive counseling and services should be offered immediately after abortion, at the site of care, as fertility can return within two weeks of the abortion (8, 9). However, several factors such as the lack of access to effective contraceptives, lack of access to PAC services, inconsistent use of short acting contraceptives, and method failure, are reported to cause a high burden of unintended pregnancies and recurrent abortion (10).

A study in Kisumu, Kenya, found that 76% of PAC clients in two public hospitals accepted post-abortion contraception (11). While systematic provision of post-abortion contraceptive counseling and services is feasible, the hospitals in the study were challenged by limited resources, shortages of trained staff and inadequate supply chain management. The researchers concluded that facilities should prioritize provider training and supervision on a wide range of contraceptive methods and make them affordable to increase access among women and adolescents. Community engagement and male involvement were recommended as strategies to support the scaling up of contraceptive uptake among PAC-seeking women.

This paper documents strategies, results, and lessons learned from Planned Parenthood Global's (PP Global) Closing the Gap Project (CTG), which aimed to improve access to and provision of comprehensive abortion care (CAC) which includes safe abortion and PAC, as well as post-abortion contraception, in South West Kenya. In addition, the CTG project sought to improve access to contraception with a focus on long-acting reversible contraception (LARC).

## Context

### Youth and Adolescent Sexual and Reproductive Health (SRH) in Kenya

Kenya has a youthful, rural population; 35.7 million Kenyans (75.1%) are below 35 years, while 32.7 million (68.9%) live in rural areas. Studies show that more than 40% of births in Kenya are unintended, rising to 47.3% among teenagers (12). Nearly half (49%) of sexually active adolescents aged 15–19 use modern contraception; 18% of 15–19 year-olds have begun childbearing (12). It is estimated that 13,000 girls drop out of school every year due to pregnancy (13). According to the Kenya Health Information System, a total of 333,812 adolescents were pregnant in 2020. The majority of those pregnant adolescents (93%) were aged between 15 to 19 years, while (7%) were aged between 10 and 14 years. On average, 23% of all pregnant mothers were adolescents aged 10 to 19 years (14). One study of adolescents presenting for PAC found that adolescents age 12–19 were more likely to seek PAC for a second trimester abortion than older women and more likely to have severe complications (15). Because adolescents in Kenya are more likely to seek late term abortion services as compared to older women, efforts to reduce unsafe abortion and its associated complications must therefore target young women; particularly adolescents.

### Abortion Context in Kenya

Many young women in Kenya who experience an unintended pregnancy resort to a unsafe abortion (16). Kenya has restrictive abortion laws and high stigma around premarital sexual activity, which further stigmatizes and impedes access to sexual and reproductive health services and effective contraception (16). Although Kenya's 2010 constitution permits abortion when a trained health care provider determines the health or the life of the woman is at risk (17), the penal code has not been updated and still criminalizes abortion, creating confusion among providers and reducing the number willing to provide safe abortion (SA) care (18). This contradiction, along with high abortion stigma (19), discourage women from seeking abortion care. Restricted access to safe abortion care because of legal prohibitions and cultural barriers is associated with increased severity of complications from abortion (20). The Kenyan Ministry of Health (MOH) recognizes PAC as one of six pillars needed to improve maternal and newborn survival; the 2019 PAC guide for providers lays out the required components of quality PAC (21). Despite this guidance, however, a major challenge in Kenya has been to reconcile rights-based approaches to providing SRH information and services with conservative approaches that oppose access to contraception, safe abortion and quality PAC, particularly for young people [aged 10–24 years old, as defined by the World Health Organization (22)]. Moreover, PAC services are not prioritized by county or national government health agendas (23). Improvements in community knowledge of abortion care, accessibility of services, and post-abortion contraception are needed to reduce unsafe abortion and prevent unintended pregnancy in Kenya (8).

In such restrictive policy and legal environments, women resort to abortions performed outside of clinical services in unsafe conditions and by unqualified providers (24). In 2012, the most recent year for which data are available, an estimated 464,690 induced abortions occurred in Kenya, and about 119,912 women sought treatment for abortion complications at a health facility (23). Forty-five percent of those receiving treatment for the most severe complications (including sepsis, fever, shock, and organ failure) were 10–19 years old. Over 70% of women and girls receiving PAC did not use any contraceptive method prior to becoming pregnant (25).

### Intervention Setting

PP Global's Closing the Gap program aimed to increase awareness of, access to, and use of CAC and contraceptive services, particularly among women and young people, in five high-need communities in Homa Bay, Kisii, Kisumu, Migori, and Siaya counties in South West Kenya. Table 1 summarizes key SRH indicators for the intervention counties, where rates of HIV and teenage pregnancy are high in comparison to the national rates.

**Table 1:** Key SRH indicators across the intervention counties and national level in Kenya.

**Indicator**	**Kisii**	**Kisumu**	**Siaya**	**Homabay**	**Migori**	**National**
Modern Contraceptive Prevalence Rate	66%	62%	56%	46%	44%	53%
Fertility rate	3.7	3.6	4.2	5.2	5.3	3.9
Proportion of mothers aged between 15 and 19 years	18%	15%	17%	33%	20.9%	15%
Median age at first birth among women age 25 - 49 years	19.5	19.6	19.4	17.7	17.6	20.3
HIV Prevalence Rate	8.0%	19.3%	23.7%	25.7%	14.7%	6.0%

*Source: Kenya Demographic and Health Survey 2014 (12)*.

### Key Programmatic Elements and Interventions

PP Global collaborated with six local service delivery organizations and a local youth advocacy network to increase access to lifesaving SA and PAC and contraception in five Kenyan counties. The project objectives were to strengthen the capacity of partner organizations to:

1. Deliver, in a continuous and sustainable manner, quality SRH services, particularly SA and PAC and contraception services for women and young people;
2. Deliver culturally and age-appropriate information on quality and comprehensive SRH to young people and women, and address stigma associated with abortion and contraception with the aim of increasing demand for, accessibility, and utilization of services, particularly SA and PAC and contraception; and
3. Advocate and create an enabling environment for SRHR, including access to and utilization of quality SA and PAC and contraception services for young people and women.

Specific attention was placed on the integration of long-acting reversible contraceptives into abortion service provision. LARCs are the most cost-effective contraceptives, and their use reduces the rate of repeat abortions (26, 27).

The project was implemented from March 2018 to August 2020 in 31 public and 49 private health facilities: 2 public country referral hospitals, 16 public and 20 private hospitals (Levels 3 and 4) and 13 public and 29 private health centers. The project's approach focused on provider training, including capacity building for quality improvement across all facility levels; infrastructure and supply chain improvement; community mobilization; tailored demand generation activities and advocacy strategies for each county; and data, monitoring and evaluation (M&E).

## Key Interventions

### Provider Training

After conducting a training needs assessment among partners, PP Global adopted existing training materials to develop a tailored and comprehensive training package on SA, PAC, and contraception. The training materials included modules on abortion values clarification and attitude transformation, methods of uterine evacuation, management of abortion complications, and post-abortion contraception including the full range of contraceptive methods. Health providers were also trained on the PP Global Quality Improvement framework and its implementation guide, which includes the following elements of quality: availability of methods and services, availability of commodities and equipment, information and counseling, staff technical competence, client centered approach, mechanism to foster continuity of care, security, monitoring and evaluation, and referral systems.

A total of 175 Kenyan health providers (1 medical doctor, 40 clinical officers and 134 nurses) received the 3-week competency-based training. Twenty additional providers were trained as mentors and trainers of trainers (TOTs) to carry out mentorship and on-the-job training across the 80 health facilities. The TOTs were drawn among the CAC and LARC trained providers that had successfully undergone the mentorship program and demonstrated a high level of competency in service provision. The selected TOTs were providers that held clinical supervisory roles at different levels to ensure they would incorporate on-the–job training into their supervisory roles beyond the project implementation period. PP Global deliberately included multiple facility levels and provider cadres to encourage peer referral and support, both within and outside of project supported sites. Refresher training was provided every six months to ensure that providers' skills were maintained. Additionally, monthly provider sharing meetings (PSM) at the facility level were held to support their continued SRH education. The objectives of PSM were to provide clinical updates based on the knowledge gaps identified by the providers and clinical mentors, strengthen team building among the providers, review quality of care implementation processes per the QOC framework, and identify challenges experienced by providers and strategies for addressing them.

Each CTG-supported health facility formed SRH quality improvement teams. At minimum, these teams were composed of the facility in-charge/manager, the lead community health volunteer (CHV), and all providers who were trained through the project. Larger facilities also included records clerks and quality assurance monitors in their teams. The teams used a quality improvement tool to assess quarterly progress at each facility and identify and address gaps.

A crucial initiative within the project's capacity building strategy was the development of the quality assessment tool. The tool includes questions that can be answered using observation, or individual or group interviews with service providers and clients. It assesses quality based on the World Health Organizations' seven pillars of health systems strengthening (28), as well as the structures and processes in place to support the domains of quality (safety, timeliness, effectiveness, efficiency, equity, and people-centeredness) (29). Following baseline assessments at each of the 80 facilities, quarterly follow up assessments were conducted at the facility and partner levels to evaluate progress. Strengths and weaknesses identified through the use of the tool were communicated back to the partners and the facilities.

In addition to facility-based SRH quality improvement teams, project mentorship units were established. The units consisted of the project partner organizations' quality improvement coordinators, representatives from the Ministry of Health Reproductive Health Unit, and PP Global quality of care technical experts, and were tasked with providing oversight of the project's mentorship processes and quality improvement teams. At the facility level, the mentorship unit conducted quarterly supportive supervision visits in collaboration with the quality improvement team at the facility using standardized checklists to assess health facility infrastructure and health workers' competencies providing SA and PAC services and PAFP. The setting up of quality improvement teams at facility level through an integrated approach meant that abortion and post abortion care contraception counseling and service provision was integrated in the health programs offered at the facilities. Quality improvement teams (QI teams) took a holistic approach to quality improvement across the entire facility health care delivery while integrating abortion and post abortion contraception. This remarkably addressed stigma around stand alone abortion services, contraception for young people and encouraged providers to be more receptive to provision of these services. These teams had leaders appointed from within the facility and thus had profound knowledge of their own systems, and could identify, test, and implement improvements to achieve the highest quality of care in their settings. The QI teams engage in regular analysis of facility process and outcome data and in quality improvement efforts driven by this data, fostering a culture of quality, motivation, and institutionalization of QI initiatives. At the provider level, newly trained providers (mentees) were assigned to mentors who were skilled providers with medical and surgical abortion care in addition to long term contraceptive technical competencies and prior experience with SRH mentorship. PP Global provided training on the project's mentorship process to all mentors, who traveled between facilities and provided technical assistance to mentees during the project period. Mentors used logbooks to track the clinical competencies of mentees who graduated from the program upon achieving full competency in LARC insertion and removal and manual vacuum aspiration (MVA).

### Health Facility Upgrades and Supplies

At the beginning of the project, a health facility assessment was conducted in each health facility to identify needs and resources. Initially, many facilities provided post abortion care (specifically, MVA) in delivery rooms. To address the inadequate space and lack of confidentiality inherent in delivery rooms, the project worked to ensure that each facility had separate procedure rooms for conducting MVA to ensure that providers had adequate space and clients received confidential care. Essential commodities (LARCs, MVA kits), supplies, equipment and medications for abotion service provision and contraception were purchased and distributed to the facilities.

The CTG project considered services to be sustainable if they have been institutionalized or incorporated into routine facility practices and therefore would likely continue after the end of external support. The physical and technical quality improvements carried out within the facilities translated into enhanced client satisfaction as identified through client exit interviews. Positive feedback from clients is a good indicator of sustainability in terms of recruiting new users and maintaining those clients who are already receiving services. By the time of closeout, all 80 facilities across were consistently reporting SA and PAC data - an indication that abortion care had been integrated into their practices. Furthermore, the collaborative work between the public and private sector resulted in multiple achievements that positively impacted the long-term sustainability of partner organizations, including enhanced commodity security and sustainability; strengthened referral networks; improved outreach and engagement of young people; and reduced abortion stigma. The project design involved a Kenyan advocacy partner organization that conducted advocacy toward increased SRHR budgetary allocation at county levels with positive results that ultimately supported SRHR training and LARC commodities.

### Engagement With the Ministry of Health and Kenyan Government

To ensure a sustainable approach to the program, the CTG project was done in close collaboration with local county health departments, the Kenyan Ministry of Health, and other governmental bodies. One key to long-term success was integrating the MOH's Reproductive Health (RH) coordinators into the project's mentorship supportive supervision structure at the start of the project. The project's mentorship structure had County Mentorship Support Units (CMSU) responsible for providing oversight, coordination, and support for each partner's mentorship activities. RH coordinators at the county level were included in CMSU and have now integrated CTG mentorship activities into their regular support supervision responsibilities — ensuring government involvement in partner facility quality improvement efforts beyond the CTG project implementation period.

PP global closely worked with the Health Information department at the MOH to ensure all public and private health facilities in the CTG project were enrolled in the national DHIS2 system. This ensured that health facilities were reporting their service delivery data on a monthly basis into the national registry. PAC and Post Abortion contraception data was shared with the MOH; safe abortion data was not shared. PP Global conducted joint routine supervision of data management, reporting systems, and data quality with the government. During the routine supervision visits, we continued to train and build the capacity of health providers and health information officers capacity to strengthen data management and reporting.

### Community and Youth Engagement

Addressing stigma around abortion was a core pilar in the project's community engagement strategies. The entry point for community engagement focused on prevention of deaths due to unsafe abortion. Through outreach to women and girls, teachers, religious leaders, and community members, the project delivered a key message: Breaking the silence around abortion is important and can save lives. At all forums, the community members were provided space to share stigma-related experiences that resulted in harm or death of young girls. This strategy sparked conversations and emphasized the harm that stigma caused. PP Global partnered with its implementing partners to sponsor “intergenerational” community dialogues, local radio programs, and workshops where participants could discuss and examine their attitudes about abortion.

PP Global trained 336 community health volunteers (CHVs) and 246 youth peer providers (YPPs) to deliver culturally and age-appropriate information on SRH to young people, and address stigma associated with abortion and contraception with the aim of increasing demand for, and accessibility and utilization of CAC and contraceptive services. For sustainability, from inception, the project engaged the CHVs that were already working within the community units under the MOH community engagement strategy. YPPs were independently mapped by the implementing partners and were paid a monthly stipend for their work. While YPP sustainability within the system was one of our biggest challenges, the YPPs built a strong network of referrals and are still referring clients for care.

With the approval of like-minded community leaders and gatekeepers such as area chiefs and church leaders, the CHVs were able to take advantage of communal gatherings like barazas (gatherings organized by village elders) to introduce themselves and the project, and conduct health talks. CHVs and YPPs also disseminated information about SA and PAC and contraception to young people at organized sports tournaments.

In order to reach young people who were unable to attend public barazas or who felt uncomfortable discussing SRH issues in public, YPPs, who were already actively involved in youth activities in these communities, visited young people in their homes where they could provide SRH information privately, and referred them to the CTG-supported facilities for youth friendly education, counseling, and clinical services. YPPs were also stationed at the project-supported facilities to provide information about youth friendly services, guide young people to the appropriate waiting areas, and provide support. This was particularly helpful at larger facilities with multiple service areas.

Through their interactions with young people, the YPPs learned that the maternal and child health (MCH) departments where RH outpatient services were provided at most facilities, and especially public facilities, were only open during general working hours (8am to 5pm), which was a barrier for young people who worked or were in school. In response, the YPPs worked with CTG project staff and the supported facilities to expand facilities' hours of operation to include evening and weekend hours.

*In the early stages of the CTG project, health providers reported that some teachers disapproved of students using contraception and forcefully sent students to health facilities to have the devices removed*. Recognizing the importance of teachers as influencers and disseminators of information, CHVs spent a considerable amount of time educating teachers on the importance of equipping students with SRHR information. Educating the teachers using values clarification and assessment transformation techniques was a useful approach for helping to shift attitudes in this key cohort.

After receiving training from PP Global on messaging and community engagement strategies, lead CHVs from each facility collaborated with the partner organizations to roll out activities aimed at increasing male involvement, including holding SRHR forums for men and identifying and mentoring male champions. These champions were attached to facilities through a team leader who provided them with SRH information and job aids to support their grassroots service demand creation work. Slowly over the course of the project, more couples started to seek SRH services in the facilities, including contraceptive sessions and SA and PAC services. The increase in couple counseling for SRH indicates rising normalization of joint decision making on SRH needs.

### Monitoring and Evaluation

PP Global developed a M&E framework for use at the partner and facility levels. Data collection tools for SA and PAC services and community engagement, and referral across facilities and from the CHVs/CHWs to facilities were developed. A standardized health facility SA and PAC register, including postabortion contraceptive uptake, was introduced in each health facility. Monthly data were collected at the facility level, aggregated, and submitted (with the exception of safe abortion data) to the Kenyan Health Information System.

## Project Results

### Community Engagement

The YPPs and CHVs conducted different types of demand creation activities during the project period. Seventy-seven SRH days were held in the health facilities to increase awareness and improve uptake of SA and PAC and contraception services. During the SRH days, the CHVs and YPPs conducted health education sessions and explained what SRH services were available in the health facilities and provided referrals to clients seeking those services. Additionally, the YPPs conducted 3,615 youth to youth sessions at youth friendly centers within the health facilities to increase awareness and utilization of SRH services. CHVs and YPPs organized 12,064 community health education sessions at the community and health facilities and 69 advocacy grassroots forums. In addition, CHVs organized 145 dialogue forums with men. The CHVs and YPPs reached 392,483 individuals with SA and PAC and contraception information from March 2018 through August 2020. The majority (62%) were adolescents and young people aged 24 years and below.

### Client Referrals and Successful Referrals

Table 2 summarizes data on client referrals and successful referrals made by CHVs and YPPs: From March 2018 through August 2020, referrals were made for 20,683 people for PAC and SA services; 67% of those referred were under the age of 25. Seventy percent of referrals were successful: 14,455 (70%) of the referred clients received PAC and SA services at the health facilities.

**Table 2:** Client referrals and successful referrals by community health volunteers and youth peer providers for safe abortion and post abortion care.

	**Adults** **(25 years or older)**	**Youth** **(24 years or younger)**	**Unknown age**	**Total**
Client referrals: safe abortion and post abortion care	6,791	13,892	67	20,683
Successful referrals: safe abortion and post abortion care	4,611	9,844	19	14,455

### Service Delivery

**Figure 1:**
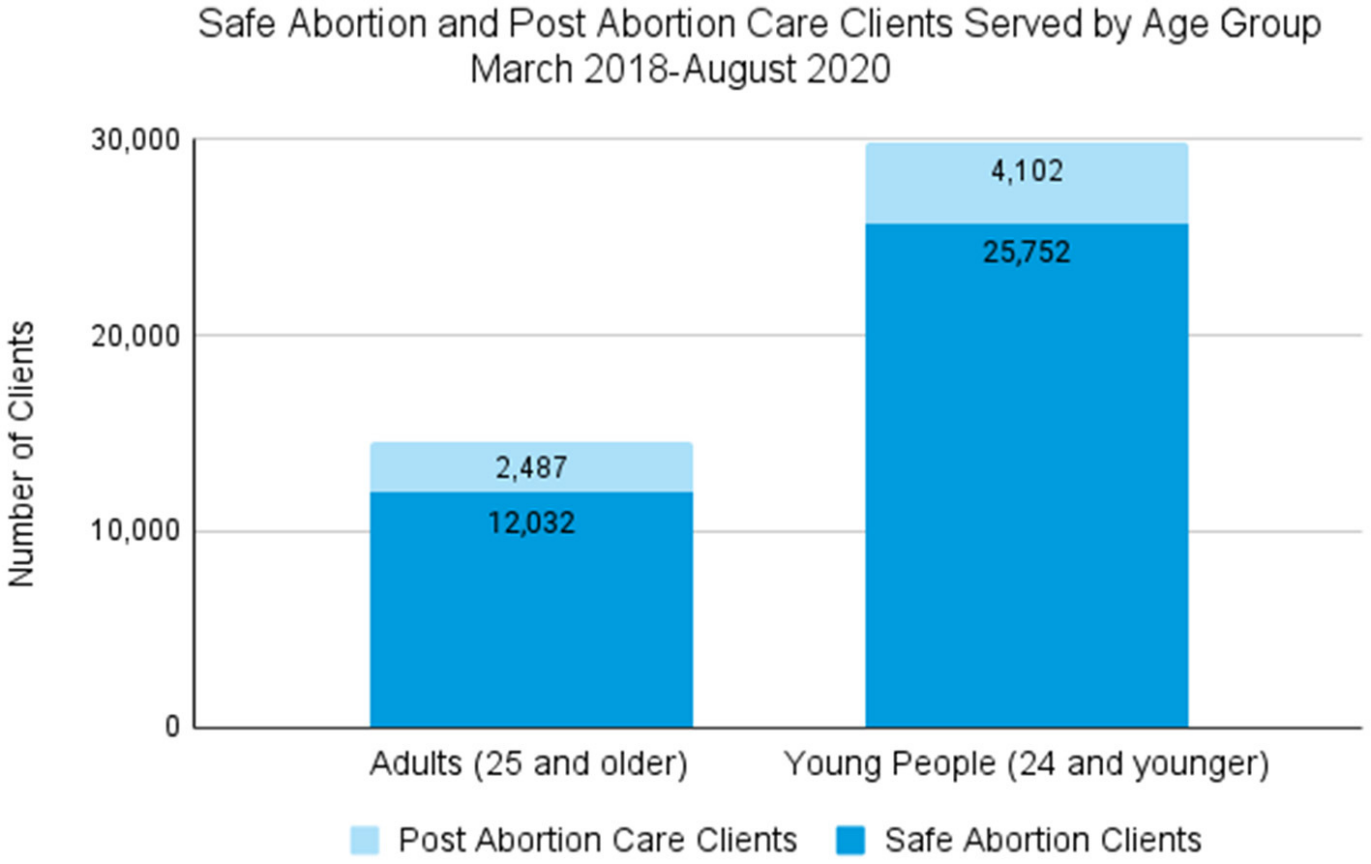
Safe abortion and post abortion care clients served by age group, March 2018 - August 2020.

From March 2018 to August 2020, 44,396 clients received safe abortion and post abortion care services; the majority (59%) of these were served in private facilities. Of the total SA and PAC clients, 85% received SA services and 15% received PAC. Seventy percent of clients had a Medical Abortion (MA), while 39% received manual vacumm aspiration (MVA). Figure 1 shows the age breakdown of SA and PAC clients; overall, two-thirds of total clients served (67%) were aged 24 years and younger.

**Figure 2:**
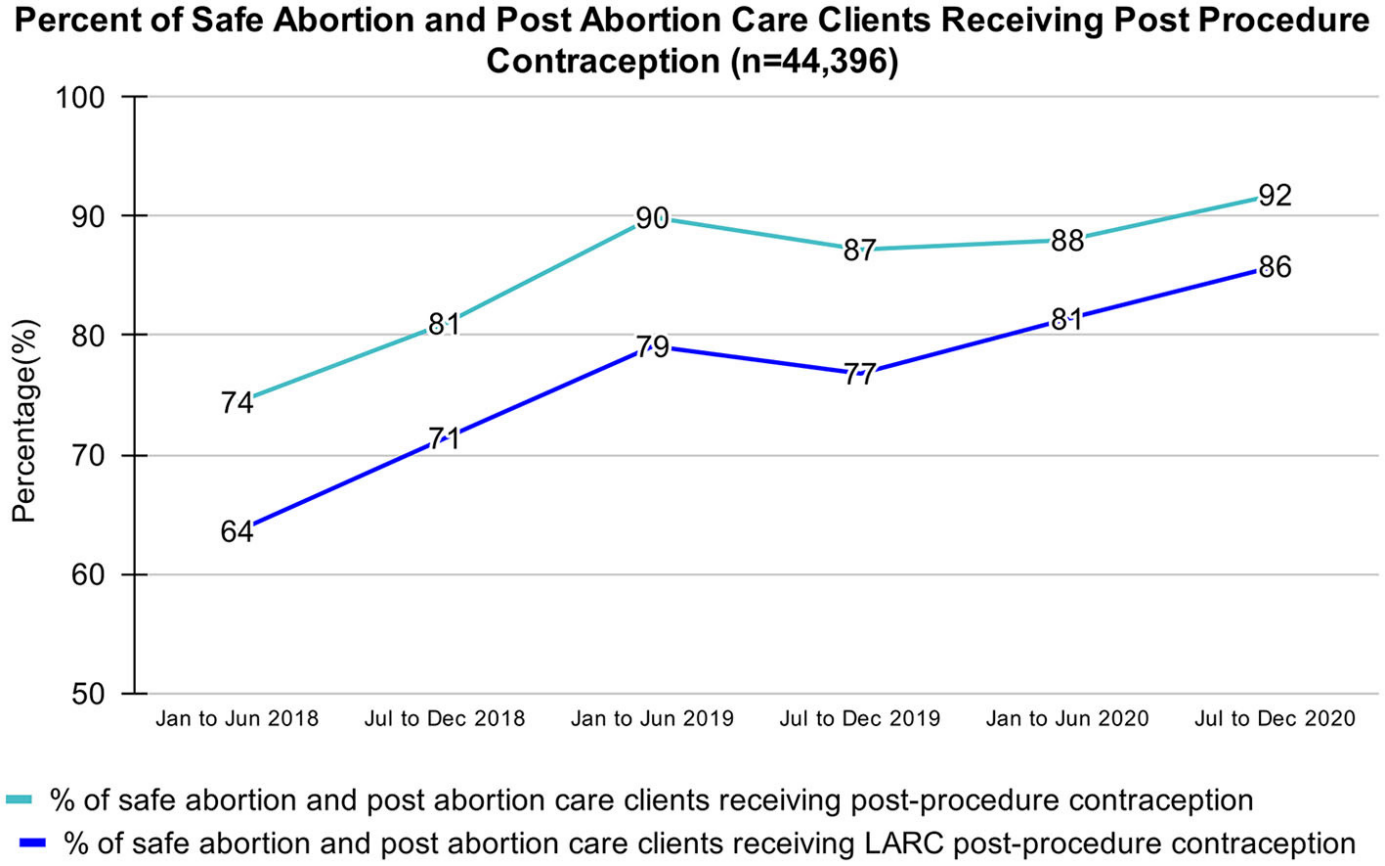
Percent of safe abortion and post abortion care clients receiving post procedure contraception, March 2018 - August 2020.

**Figure 3:**
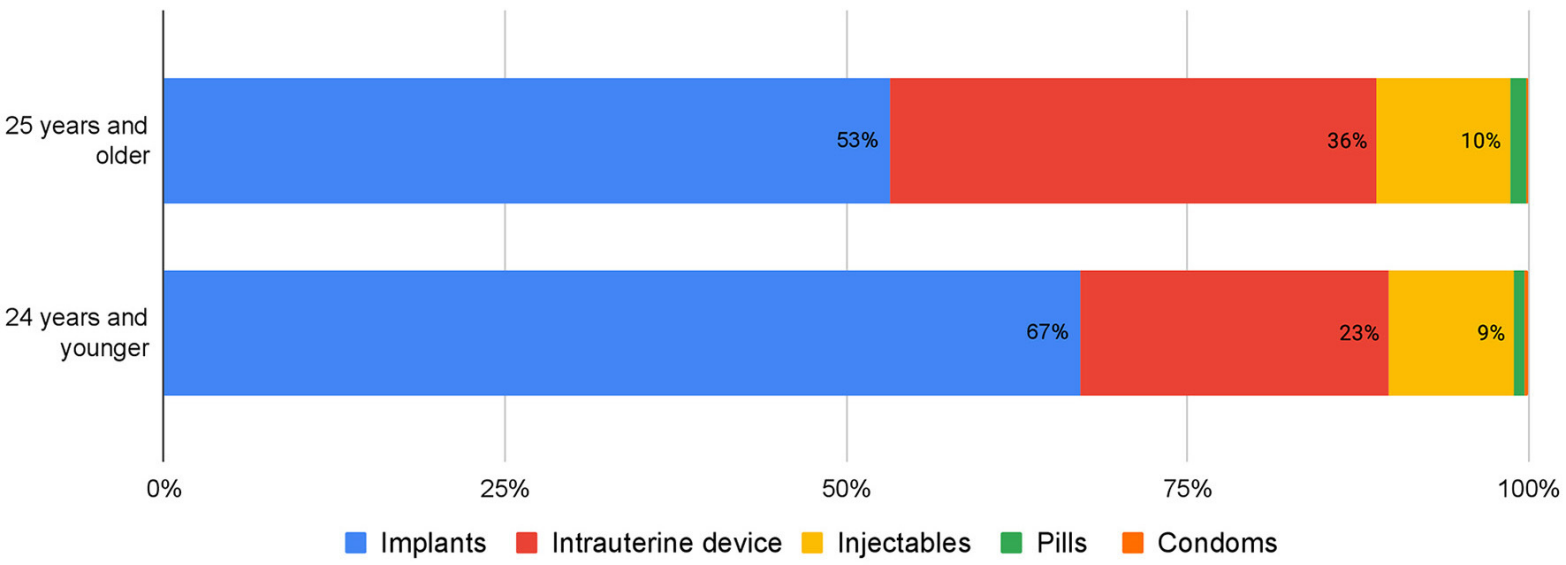
Post-abortion contraception clients, by age and contraceptive method received, March 2018 - August 2020.

Overall, the majority of SA and PAC clients (85%) accepted post-procedure contraception. Figure 2 details the trends in the uptake of this service. From January 2018 to December 202, the percentage of SA and PAC clients who received a post-procedure contraception rose, from 74 to 92%. Likewise, the percentage of safe abortion and post abortion care clients who received a post-procedure LARC rose from 64 to 86%. A further disaggregated method mix of post-procedure clients who chose a contraceptive is shown in Figure 3; the vast majority of both young people (90%) and adults (89%) chose a LARC.

## Discussion

Post-abortion contraception is one of several High Impact Practices in Family Planning (HIPs) identified by global SRH experts (10), yet expansion has been challenging in Kenya. Our experience implementing the CTG project in South West Kenya shows that training and mentorship of mid-level providers on SA and PAC, post-abortion contraception and youth friendly services, coupled with community engagement and referral, removes common barriers to care for women and young people. In turn, this multi-pronged intervention likely contributed to reductions of unintended pregnancies and unsafe abortion.

The CTG project was successful in reaching young people with SA, PAC, and contraception information, referrals, and services: over the three-year project period, two-thirds of SA and PAC clients served were aged 24 years or younger. Prior research on adolescent SRH service utilization and access in Kenya has found that negative provider attitudes are a major barrier to care, particularly for abortion-seeking adolescents (30, 31). In the CTG project, all facilities were equipped to deliver youth friendly services. Our results indicate that values clarification, as well as specialized training of providers and post-training mentorship on youth friendly care should be integrated into future facility-based interventions aimed at increasing SA and PAC provision. In addition, expanding facility hours to include evenings and weekends, and stationing YPPs at facilities to provide information about and direct young people to desired services, were particularly effective strategies for facilitating young people's use of SA, PAC, and contraception.

An important aim of the CTG project was to integrate LARCs into SA and PAC service provision. Eighty-five percent of SA and PAC clients served by the project accepted same-day contraception, with the vast majority—including 90% of clients aged 24 and under—receiving LARCs. This represents a departure from other research findings on post-abortion contraceptive uptake in low- and middle-income countries, including Kenya, where more clients received short-acting methods (32–34), and providers reported inadequate counseling time available to cover LARCs (35). Similarly, prior to the start of the CTG intervention, providers working in supported health facilities were hesitant to counsel on and administer LARCs to adolescent or youth clients. CTG training and mentoring addressed this issue. In response to this and a growing body of literature on the effect of contraceptive counseling on uptake of post abortion contraception (11, 33, 36, 37), CTG trained providers to use a time allocation strategy for contraceptive counseling, whereby 70% of counseling time is spent on LARCs, and 30% is spent on short acting methods (38). Alongside ongoing provider mentorship, implementation of this timing strategy enabled providers to grow their capacity and confidence to administer LARC services.

The project's success at increasing utilization of SA and PAC and LARCs was also driven by its focus on community-based demand-creation activities. Education and referrals provided by YPPs and CHVs raised awareness of and linkage to facilities providing these services, helped change community attitudes, and reduced stigma associated with abortion and contraception. The effectiveness of community intermediaries in raising awareness and utilization of SA and PAC has been observed in other countries as well (39–41).

The stigma surrounding safe abortion services is an ongoing challenge. Initially the project implementing partners were hesitant to be openly supportive of increasing access to safe abortion. In addition, providers expressed concerns about providing abortion services — fearing intimidation, harassment, or even prosecution under restrictive laws around safe abortion access and lack of clear safe abortion care policies and guidelines within the Ministry of Health. PP Global worked on several fronts to help increase the number of trained providers for competency is safe abortion provision and increase access to this critical service. Along with promoting positive, stigma-free communication and information sharing around safe abortion at the community level, PP Global worked to counter negative stereotypes, dispel myths, provide clarity about abortion laws and policies, and empower partners to provide abortion care across the project-supported facilities. The result is that more trained providers in both public and private facilities can provide quality abortion care, and women and young people can access safe abortion without stigma or discrimination. At the project close-out, online forums for providers remain active, and users have reported increased provider knowledge and reduced stigma around abortion service provision.

Finally, the incorporation of a strong SRHR advocacy partner into this service delivery project provided the opportunity to build networks of like-minded individuals and organizations to advocate for and enhance access to contraception and safe abortion. In particular, youth-led SRHR advocacy groups should be prioritized in future projects to give voice to the young people who have long been denied the opportunity to make informed decisions about their sexual and reproductive health.

## Conclusion

This case study demonstrates the success of the CTG project to expand provision of SA and PAC in South West Kenya, and encourage utilization of SA, PAC, and post abortion contraception, especially among young people. Pairing health facility improvements and provider training with targeted community engagement activities, including specific outreach and support for youth and adolescents, contributed to this success. This approach carries the potential to accelerate progress toward the government's goal to expand equitable access to quality contraception at the county level (42). CTG's success suggests that improvements to safe abortion care and contraceptive service delivery, combined with targeted community outreach, will help young people and women achieve their reproductive goals.

## Data Availability Statement

The raw data supporting the conclusions of this article will be made available by the authors, without undue reservation.

## Author Contributions

All authors listed have made a substantial, direct and intellectual contribution to the work, and approved it for publication.

## Funding

The Closing the Gap project was funded by an anonymous donor.

## Conflict of Interest

The authors declare that the research was conducted in the absence of any commercial or financial relationships that could be construed as a potential conflict of interest.

## Publisher's Note

All claims expressed in this article are solely those of the authors and do not necessarily represent those of their affiliated organizations, or those of the publisher, the editors and the reviewers. Any product that may be evaluated in this article, or claim that may be made by its manufacturer, is not guaranteed or endorsed by the publisher.
